# Interaction Between Peripheral Cytokines and Central Neuroinflammation in Temporal Lobe Epilepsy

**DOI:** 10.1002/cns.71200

**Published:** 2026-10-10

**Authors:** Heyu Zhang, Ningning Wang, Yuying Zhang, Siqi Ou, Jingjing Chen, Jinming Zhang, Yicong Liu, Yuting Luo, Yingting Luo, Ziheng Wei, Kailin Wu, Xiangsong Zhang, Ziyi Chen, Guanzhong Ni

**Affiliations:** ^1^ Department of Neurology The First Affiliated Hospital, Sun Yat‐Sen University, Guangdong Provincial Key Laboratory of Diagnosis and Treatment of Major Neurological Diseases, National Key Clinical Department and Key Discipline of Neurology Guangzhou China; ^2^ Department of Nuclear Medicine The First Affiliated Hospital, Sun Yat‐Sen University Guangzhou China; ^3^ Department of Neurosurgery The First Affiliated Hospital, Sun Yat‐Sen University Guangzhou China

**Keywords:** neuroinflammation, PET/MRI, serum cytokines, temporal lobe epilepsy, translocator protein

## Abstract

**Purpose:**

Neuroinflammation is a key pathogenic mechanism in temporal lobe epilepsy, quantifiable in vivo using positron emission tomography targeting the translocator protein. This study integrated [^18^F]DPA‐714 PET/MRI with serum cytokine profiling to investigate this central–peripheral immune relationship in TLE patients.

**Methods:**

A prospective study of 24 TLE patients and 24 healthy controls (HCs) was conducted. PET/MRI data were available for 24 TLE patients and 7 HCs, while cytokine data were available for all 24 participants in both groups. Standardized uptake value ratios of [^18^F]DPA‐714 were calculated for key temporal lobe regions. Serum levels of IL‐2, IL‐4, IL‐6, IL‐10, IL‐17, TNF‐α, and IFN‐γ were quantified.

**Results:**

Compared with HCs, TLE patients demonstrated significantly increased [^18^F]DPA‐714 PET/MRI uptake in ipsilateral temporal lobe structures (group main effect: *F*_1,29_ = 15.779, *p* < 0.001), accompanied by a proinflammatory serum profile with elevated IL‐17 and IFN‐γ and decreased IL‐2, IL‐4, and IL‐10 levels (*p* < 0.05). Ipsilateral hippocampal [^18^F]DPA‐714 SUVR was positively correlated with disease duration (*ρ* = 0.46, *p* = 0.025) and seizure frequency (*ρ* = 0.52, *p* = 0.018). However, no significant associations were observed between neuroimaging‐assessed central neuroinflammation and serum cytokines.

**Conclusion:**

[^18^F]DPA‐714 PET/MRI precisely demonstrates focal neuroinflammation in TLE patients, correlating with clinical severity, yet the lack of correlation with peripheral markers highlights that peripheral and central immune responses represent distinct and relatively independent inflammatory processes in TLE.

## Graphical abstract

[^18^F]DPA‐714 PET/MRI revealed significantly increased uptake in ipsilateral temporal lobe structures of TLE patients. Ipsilateral hippocampal SUVR positively correlated with disease duration and seizure frequency. Serum cytokine profiling showed elevated IL‐17 and IFN‐γ and decreased IL‐2, IL‐4, and IL‐10 levels in TLE patients, but no significant correlations were found between central neuroinflammation and serum cytokines.

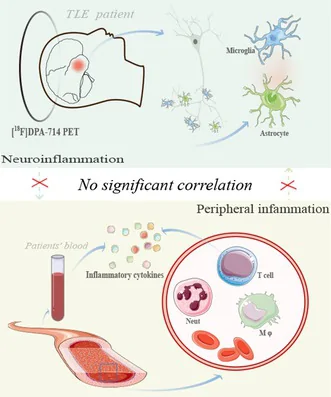

## 1 Introduction

Temporal lobe epilepsy (TLE) is the most common type of focal epilepsy in adults, accounting for approximately 20%–40% of cases of drug‐resistant epilepsy [1]. Existing perspectives suggest that neuroinflammation is both a cause and a consequence of epilepsy. Studies on animal models and postsurgical pathology in patients have shown that neuroinflammatory reactions play a significant role in the occurrence and progression of TLE. MRI remains the usual structural imaging modality [2]. However, it fails to identify abnormalities in 20%–30% of TLE cases, largely due to subtle or nonstructural pathologies such as active neuroinflammation.

PET/MRI has emerged as a valuable imaging modality for assessing neuroinflammation in TLE patients, particularly when conventional MRI findings are inconclusive [3]. A key molecular target for imaging neuroinflammation is the translocator protein (TSPO), which is upregulated on the outer mitochondrial membranes of activated microglia and reactive astrocytes, making it a reliable biomarker of neuroinflammation. The use of TSPO‐targeted tracers, such as [^18^F]DPA‐714, enables noninvasive visualization of microglial activation, a central process in epilepsy‐associated neuroinflammation [4, 5].

Complementing these neuroimaging advances, significant research has explored the identification of peripheral biomarkers such as inflammatory cytokines in patients with TLE. Studies have shown that during the interictal period, TLE patients exhibit elevated levels of peripheral inflammatory markers, which display dynamic temporal changes following seizures [6, 7]. Specifically, within 24 h postseizure, the levels of proinflammatory cytokines, including interleukin‐1β (IL‐1β), IL‐6, and tumor necrosis factor‐α (TNF‐α), peak and are significantly higher in patients with TLE than in healthy controls [8, 9]. Although the levels of the majority of markers exhibit a decline following the occurrence of seizures, the IL‐1β level remains elevated up to 1 month, thereby indicating the potential for chronic inflammation to persist [10]. Collectively, these findings indicate that inflammatory cytokines are key contributors to TLE pathophysiology, offering a promising approach for the dynamic monitoring of the disease course [11].

Current research gaps include elucidating causal links between PET/MRI‐derived inflammatory signatures and peripheral biomarkers and leveraging multimodal data for personalized therapeutic strategies. This study aims to integrate [^18^F]DPA‐714 PET/MRI with serum cytokine profiling to establish a novel biomarker framework for TLE, advancing both diagnostic precision and targeted anti‐inflammatory interventions.

## 2 Materials and Methods

### 2.1 Participants

We prospectively recruited patients who were diagnosed with TLE at the First Affiliated Hospital of Sun Yat‐sen University between July 2023 and July 2025. The diagnosis of TLE was established by at least two experienced epileptologists through comprehensive multimodal evaluation, including neurological history and examination, interictal and ictal scalp video‐EEG, high‐resolution 3 T MRI with epilepsy‐specific sequences, and [^18^F]FDG PET/MRI imaging. The diagnosis was confirmed when the findings were concordant with seizure origin from the temporal lobe. Seizure types were classified according to the International League Against Epilepsy (ILAE) 2025 operational classification [12]. Healthy controls (HCs) were enrolled from the Health Examination Center of the same hospital and matched 1:1 to the TLE patients by sex and age; seven of these controls also underwent PET/MRI.

The patient inclusion criteria were as follows: (1) age between 16 and 65 years; (2) diagnosed with either definite or probable TLE as defined above; (3) availability of complete structural MRI, [^18^F] FDG PET/MRI, and [^18^F]DPA‐714 PET/MRI data; and (4) ability to comprehend the study objectives, cooperate with the research procedures, and provide written informed consent.

The exclusion criteria were as follows: (1) history of craniocerebral trauma, infectious diseases (e.g., encephalitis, meningitis), tumors, or other central nervous system disorders; (2) poor imaging quality (e.g., significant motion artifacts); and (3) low‐affinity (LA)‐binding phenotype for TSPO due to the rs6971 polymorphism.

### 2.2 Clinical Assessment

Systematic clinical data collection was performed for all participants. In the TLE cohort, this included (1) demographic characteristics (age, sex); (2) epilepsy‐specific parameters defined according to ILAE criteria—including seizure frequency, seizure type, epilepsy duration, and drug resistance status; and (3) the critical time interval between the last seizure and the [^18^F]DPA‐714 PET/MRI scan. For HCs, data collection was limited to demographic characteristics (age and sex).

### 2.3 PET/MRI Acquisition

Participants underwent simultaneous PET/MRI acquisition on a 3.0‐T hybrid PET/MRI scanner (uPMRI790; United Imaging Healthcare) with a head and neck coil. PET acquisition began 60 min after intravenous injection of [^18^F]DPA‐714, and brain MRI scans were acquired simultaneously. The sequences included a three‐dimensional T1‐weighted gradient‐echo sequence (repetition time [TR]/echo time [TE] = 7.9/3 ms, inversion time [TI] = 750 ms, flip angle = 10°, field of view [FOV] = 230 mm × 256 mm, and voxel size = 1 mm × 1 mm × 1 mm), a three‐dimensional T2‐weighted fluid‐attenuated inversion recovery (FLAIR) sequence (TR/TE/TI = 4800/382.12/1594 ms, FOV = 240 mm × 240 mm, and voxel size = 1 mm × 1 mm × 1 mm), a high‐resolution coronal T2‐weighted fast spin–echo sequence of the hippocampus (TR/TE = 7128/105.2 ms, flip angle = 90°/120°, 6 averages, FOV = 180 × 180 mm, and voxel size = 0.59 mm × 0.47 mm × 2 mm), and a coronal T2‐weighted FLAIR sequence of the hippocampus (TR/TE/TI = 7508/114.92/2340 ms, flip angle = 90°/150°, 6 averages, FOV = 180 mm × 180 mm, and voxel size = 0.94 mm × 0.7 mm × 2 mm). PET images were reconstructed using an iterative algorithm (HYPER Iterative; *β* = 0.28) with a 256 × 256 matrix, a 300‐mm field of view, and a 2‐mm slice thickness.

### 2.4 Image Analysis

Anatomical T1‐weighted MR images were processed using the FreeSurfer recon‐all pipeline (version 7.4.1; http://surfer.nMRI.mgh.harvard.edu) with the Desikan–Killiany atlas. Reconstructed PET images were aligned and coregistered to the processed T1‐weighted MR images for each subject. The PETsurfer pipeline was employed for partial volume correction (PVC). This pipeline sampled the corrected PET data onto the individual's cortical surface, applied geodesic smoothing (6‐mm FWHM kernel), and resampled the data onto the fsaverage template.

Atlas‐derived regions of interest (ROIs) were applied to the processed images to extract regional volumes and SUVR. The whole cerebellar gray matter was used as the reference region for SUVR normalization [13]. To reduce the burden of multiple comparisons, Desikan–Killiany‐defined temporal cortical parcels were merged per hemisphere. The final analysis targeted eight bilateral ROIs: hippocampi, amygdalae, merged temporal cortices, and temporal poles. For TLE patients, the ipsilateral hemisphere was defined as the side of the epileptogenic focus. This localization was determined based on concordant evidence from structural MRI and PET imaging, with additional support from EEG when available. When EEG data were absent, the lateralization was determined by structural MRI findings alone. In the HCs, the left hemisphere was designated as the ipsilateral hemisphere, and the right hemisphere was designated as the contralateral hemisphere [14, 15]. The asymmetry index (AI) for [^18^F]DPA‐714 uptake was calculated from the SUVR values using the formula AI = [200% × (ipsilateral − contralateral)/(ipsilateral + contralateral)].

### 2.5 Serum Cytokines Measurement

Blood samples were obtained on the morning of the [^18^F]FDG PET/MRI examination after an overnight fast (interval to [^18^F]DPA‐714 PET/MRI: median 2 days; range: −4 to +8 days; negative values indicate blood collection after the scan). The samples were centrifuged to isolate serum, aliquoted, and stored at −80°C until further analyses. Serum cytokine profiling (IL‐2, IL‐4, IL‐6, IL‐10, IL‐17, TNF‐α, and IFN‐γ) was performed using a multiplexed cytometric bead array kit (Jiangxi Cellgene Biotech Co. Ltd., Nanchang, China) with FCAP Array Software v3.0. All cytokine concentrations were measured in pg/mL, and the lower limits of detection ranged from 0.07 to 2.5 pg/mL across analytes.

### 2.6 Statistical Analysis

All statistical analyses were performed using R software (version 4.4.2). Demographic and clinical characteristics were summarized using descriptive statistics with graphical visualization. The normality of continuous variables was assessed using the Shapiro–Wilk test. Group comparisons between healthy controls and temporal lobe epilepsy patients employed independent‐ samples *t*‐tests for normally distributed variables, Mann–Whitney *U* tests for non‐normally distributed data, and chi‐square tests for categorical variables (e.g., sex). Multiple comparisons were adjusted using the Benjamini–Hochberg procedure to control the false discovery rate (FDR). All correlations were evaluated using Spearman's rank correlation coefficient (*ρ*). Statistical significance was defined as a two‐tailed *p* < 0.05, with effect sizes reported for significant results.

## 3 Results

### 3.1 Demographic and Clinical Characteristics

We identified 54 patients who met the diagnostic criteria for suspected TLE. A total of 30 patients were excluded based on the exclusion criteria (Figure 1). The study included 24 TLE patients and 24 HCs (mean age: 35.54 ± 10.57 years; 13 males, 54.17%), with no significant differences between the two groups in age (*p* = 0.733) or sex (*p* = 1.000). All TLE patients and the 7 HCs who underwent PET/MRI were high‐affinity binders based on rs6971 genotyping. Within the TLE group, the mean age at epilepsy onset was 25.83 ± 16.39 years, with a median symptom duration of 9 years (IQR: 5–13 years). Patients received a median of two antiseizure medications (ASMs, IQR: 2–3). With respect to focal seizure types, the majority of patients experienced focal impaired consciousness seizures (FICs) (75.00%) and focal‐to‐bilateral tonic–clonic seizures (FBTCs) (70.83%), whereas focal preserved consciousness seizures (FPCs) occurred in 26.32% of the patients. Video‐electroencephalography (VEEG) was performed in 19 patients (79.17%). Stereoelectroencephalography (SEEG) was conducted in four patients. Hippocampal sclerosis (HS) was identified on MRI in 13 patients (54.2%) and was unilateral in 12 patients and bilateral in one patient. Drug‐resistant epilepsy was observed in 50% of the cohort. Nine patients (37.50%) underwent epilepsy surgery, and among the seven patients with at least 1 year of follow‐up, all achieved an ILAE Class 1 outcome (Table 1). [^18^F]FDG‐PET hypometabolism was localized solely to the temporal lobe in 15 patients (62.5% of patients scanned) and involved both temporal and extratemporal regions in eight patients (33.3%). One patient (4.2%) had a negative [^18^F]FDG‐PET scan. Concordance among [^18^F]FDG‐PET, VEEG, and MRI results was observed in 52.63% of the patients who underwent VEEG (10/19 patients). Abnormally increased [^18^F]DPA‐714 binding was significantly more frequent in patients. It was restricted to the temporal lobe in 21 patients (87.5%) and extended to both the temporal and extratemporal regions in three patients (12.5%), and no patients had a negative scan. The concordance among the [^18^F]DPA‐714 PET, VEEG, and MRI results was significantly higher, occurring in 78.95% of patients who underwent VEEG (15/19 patients). Complete concordance across all modalities ([^18^F]FDG‐PET, [^18^F]DPA‐714 PET, VEEG, and MRI) was found in 52.63% of patients with VEEG data (10/19 patients) (Table 2). PET and MRI scans of the HCs were negative.

**FIGURE 1:**
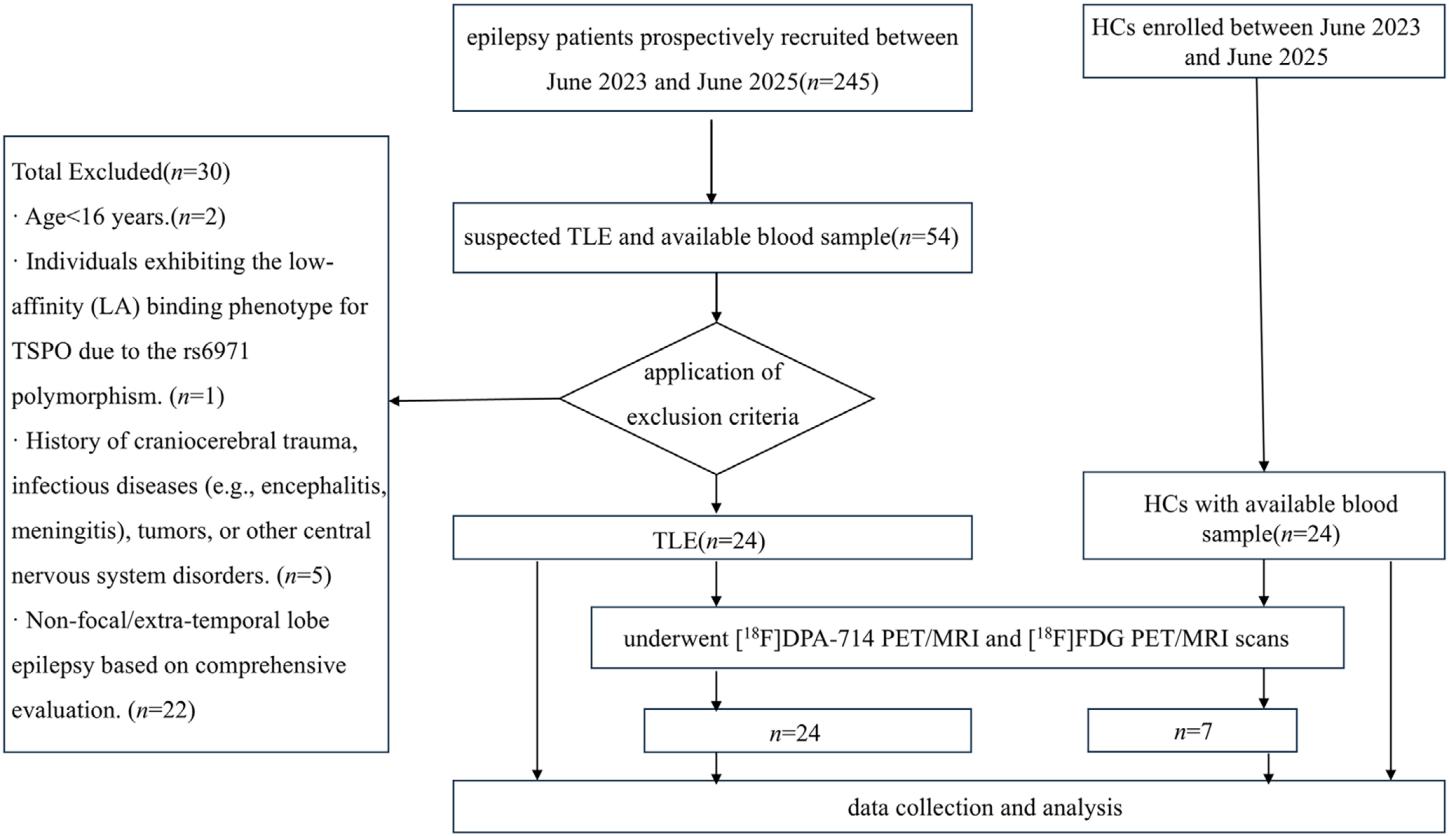
Flowchart of inclusion and exclusion criteria. The study cohort was derived from 245 epilepsy patients with complete multi‐modal PET data ([^18^F]DPA‐714 and [^18^F]FDG). Through systematic screening, two matched groups were established: 24 patients with comprehensively evaluated TLE and 24 HCs. The specific reasons for the exclusion of 30 subjects are delineated in the chart. HCs, healthy controls; TLE, temporal lobe epilepsy.

**TABLE 1:** Characteristics of patients (*N* = 24).

	Patients (*n* = 24)
Age, mean (SD), years	37.13 (13.12)
Male, *n* (%)	13 (54.17%)
Age at onset of epilepsy, mean (SD), years	25.83 (16.39)
Duration of symptoms, median (IQR), years	9 (5–13)
Number of ASMs, median (IQR)	2 (2–3)
Any focal seizures	
focal preserved consciousness seizure (FPC)	5 (26.32%)
focal impaired consciousness seizure (FIC)	18 (75.00%)
focal‐to‐bilateral tonic–clonic seizure (FBTC)	17 (70.83%)
VEEG results	
NO. recorded cases, *n* (%)	19 (79.17%)
lctal VEEG recorded, *n*	11
interictal VEEG only, *n*	8
MRI results	
NO. recorded cases, *n* (%)	24 (100%)
unilateral HS	12
bilateral HS	1
others	11
Drug‐resistant epilepsy, *n* (%)	12 (50%)
Surgical patients, *n* (%)	9 (37.50%)
Surgical outcome (ILAE Class 1), *n*	7

*Note:* Q1, 25th percentile; Q3, 75th percentile.

Abbreviations: ASMs, Antiseizure medication; HS, hippocampal sclerosis; ILAE, International League Against Epilepsy; SD, standard deviation; VEEG, vedioelectroencephalography.

**TABLE 2:** Neuroimaging findings and inter‐modality concordance analysis (*n* = 24).

	[^18^F]FDG PET (*n* = 24)	[^18^F]DPA‐714 PET (*n* = 24)	*p*
Lesion localization, *n* (%)			
temporal lobe only	15 (62.5%)	21 (87.5%)	**0.031** * ^a^
temporal + extratemporal	8 (33.3%)	3 (12.5%)	NA^b^
negative	1 (4.2%)	0 (0.0%)	
Concordance with VEEG & MRI	*n* = 19	*n* = 19	
Concordant cases, *n* (%)	10 (52.63%)	15 (78.95%)	0.062^a^

Abbreviations: FDG, fluoro‐deoxyglucose; DPA‐714, N,N‐diethyl‐2‐(2‐(4‐(2‐[18F]fluoroethoxy)phenyl)‐5,7‐dimethylpyrazolo[1,5‐a]pyrimidin‐3‐yl)acetamide. VEEG, video‐electroencephalography.

^a^ *p* values for paired comparisons were calculated using the exact binomial test.

^b^ The *p*‐value for “temporal + extratemporal” is omitted as the primary comparison is for “temporal lobe only” detection.

* *p* < 0.05.

### 3.2 [ ^18^F]DPA‐714 Uptake in Patients With TLE and HCs

Quantitative analysis of neuroinflammation, measured by [^18^F]DPA‐714 PET/MRI SUVR, revealed significant alterations in key regions implicated in disease pathology, including the hippocampus, amygdala, temporal cortex, and temporal pole. Comparisons between 24 patients with TLE and 7 HCs revealed significantly higher SUVR values in the ipsilateral ROIs (group main effect: *F*_1,29_ = 15.779, *p* < 0.001) compared with the controls, while no such difference was observed in the contralateral regions (*F*_1,29_ = 0.366, *p* = 0.550). Post hoc analysis further confirmed that within the ipsilateral hemisphere, [^18^F]DPA‐714 PET/MRI SUVR values were elevated by 28%–70% across the four temporal regions in patients compared with controls (all uncorrected *p* < 0.001). In addition to temporal regions, we also examined [^18^F]DPA‐714 PET/MRI SUVR in the bilateral thalami. No significant differences were observed between TLE patients and HCs (ipsilateral thalamus: *p* = 0.208; contralateral thalamus: *p* = 0.459) (Figure 2).

**FIGURE 2:**
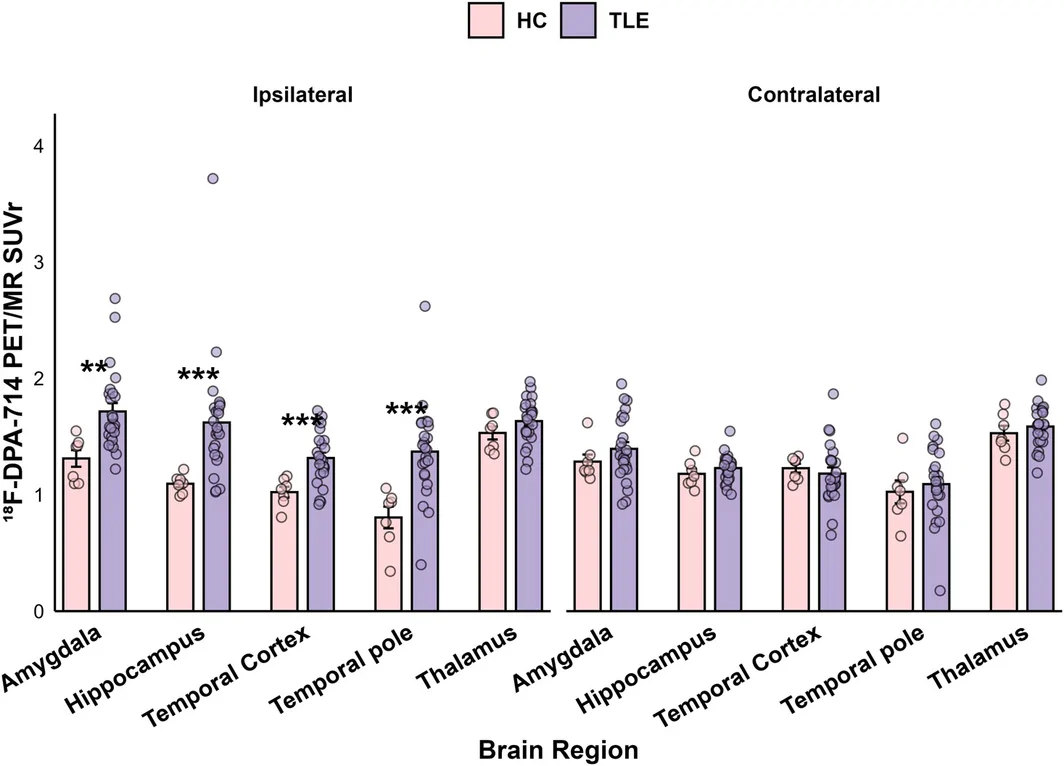
Increased [^18^F]DPA‐714 uptake in ipsilateral temporal lobes of TLE patients. Bar graph shows representative data of [^18^F]DPA‐714 SUVR in bilateral ROIs from patients with TLE and HCs. Error bars indicate standard deviation. As detailed in the text, TLE patients showed significantly higher uptake in specific ipsilateral temporal regions (by independent‐samples *t*‐test, ***p < 0.001, uncorrected). No significant thalamic differences were found between groups (ipsilateral: *p* = 0.208; contralateral: *p* = 0.459). HCs, healthy controls; ROIs, temporal regions of interest; SUVR, standardized uptake value ratio; TLE, temporal lobe epilepsy.

### 3.3 Serum Cytokines in TLE Patients and HCs

We measured and compared the serum levels of multiple cytokines (including IL‐2, IL‐4, IL‐6, IL‐10, TNF‐α, IFN‐γ, and IL‐17) in 24 patients with TLE and 24 HCs. All cytokine data were log_10_‐transformed prior to analysis. Serum concentrations of IFN‐γ (0.41 ± 0.18 vs. 0.34 ± 0.10; *p* < 0.05) and IL‐17 (0.48 ± 0.20 vs. 0.20 ± 0.12; *p* < 0.001) were significantly higher in TLE patients than in HCs. By contrast, the concentrations of IL‐2 (0.09 ± 0.18 vs. 0.30 ± 0.16; *p* < 0.001), IL‐4 (0.09 ± 0.16 vs. 0.24 ± 0.13; *p* < 0.001), and IL‐10 (0.57 ± 0.13 vs. 0.74 ± 0.13; *p* < 0.001) were significantly lower in the TLE patient group. No statistically significant differences were observed between the groups in the levels of TNF‐α (0.40 ± 0.11 vs. 0.47 ± 0.11; *p* = 0.08) or IL‐6 (0.62 ± 0.28 vs. 0.63 ± 0.16; *p* = 0.95) (Figure 3).

**FIGURE 3:**
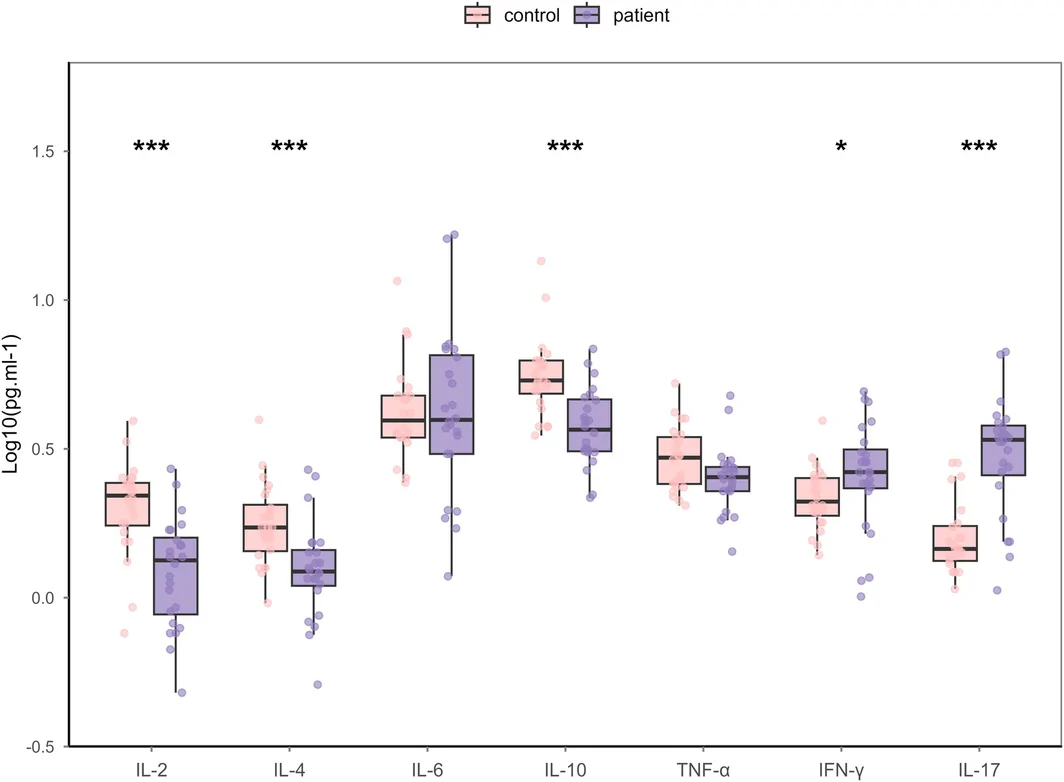
Cytokines of patients with TLE and HCs. Box plots show the comparison of cytokine levels between TLE and HCs. The boxes represent the median and interquartile range. After correction for multiple comparisons using the FDR method, specific biomarkers showed significant alterations in the TLE group (***p < 0.001, *p < 0.05). FDR, false discovery rate; HCs, healthy controls; TLE, temporal lobe epilepsy.

### 3.4 Correlation Analysis of Seizure Characteristics and [ ^18^F]DPA‐714 PET

To further investigate disease characteristics in patients with TLE, we assessed the associations of two key clinical indicators, seizure frequency and disease duration, with neuroinflammation, as assessed using [^18^F]DPA‐714 PET/MRI SUVR in the ipsilateral hippocampus. First, we analyzed the relationship between seizure frequency, defined as the total number of seizures in the month preceding the [^18^F]DPA‐714 PET/MRI scan, and the ipsilateral hippocampal SUVR. Four patients were excluded because of incomplete clinical seizure records, resulting in a final cohort of 20 patients for this analysis. Spearman's correlation analysis revealed a statistically significant positive correlation between these two variables (*ρ* = 0.52, *p* = 0.018) (Figure 4a). Second, we examined the correlation between epilepsy duration (years) and ipsilateral hippocampal SUVR, for which all 24 patients were included in the analysis. This analysis also revealed a significant positive correlation (*ρ* = 0.46, *p* = 0.025) (Figure 4b). To determine whether this association was specific to the hippocampus, we further examined the correlations between clinical severity measures and [^18^F]DPA‐714 SUVR in other ipsilateral temporal ROIs, including the amygdala, temporal cortex, and temporal pole. Neither seizure frequency nor disease duration was significantly correlated with SUVR in these regions (all *p* > 0.05) (Figure S1).

**FIGURE 4:**
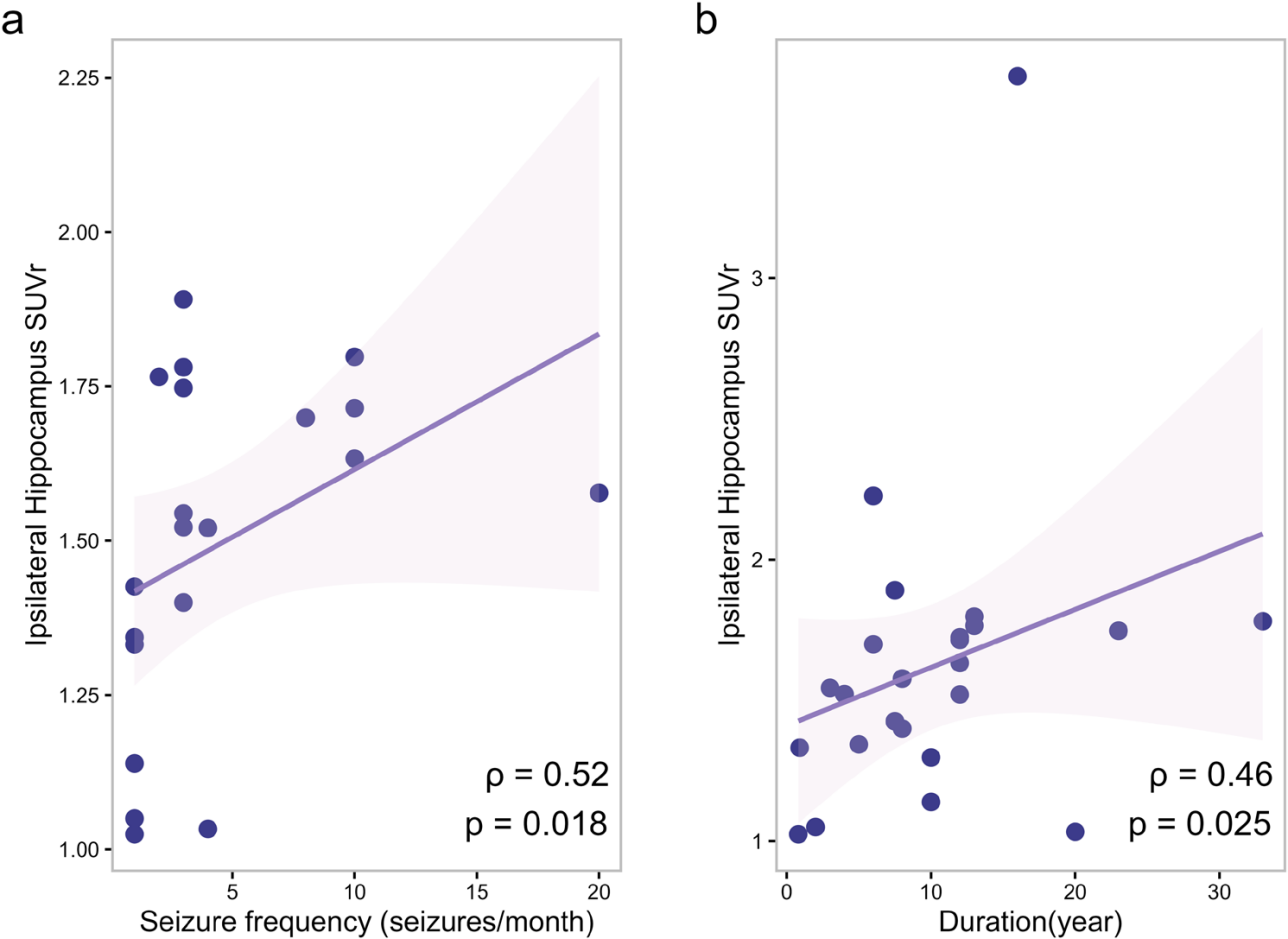
Correlations of ipsilateral hippocampal [^18^F]DPA‐714 SUVR with seizure characteristics. Relationships between the SUVR in the ipsilateral hippocampus and clinical measures were assessed using Spearman rank‐order correlation analysis. (a) Hippocampal SUVR showed a significant positive correlation with monthly seizure frequency (*ρ* = 0.52, *p* = 0.018). (b) Hippocampal SUVR showed a significant positive correlation with disease duration (*ρ* = 0.46, *p* = 0.025). All *p* values are uncorrected for multiple comparisons. SUVR, standardized uptake value ratio.

### 3.5 Correlation Between Serum Cytokines and [ ^18^F]DPA‐714 PET

To further investigate the relationship between peripheral and central neuroinflammation in patients with TLE, we analyzed the correlation between neuroinflammatory imaging markers ([^18^F]DPA‐714 SUVR) across eight ROIs and multiple serum inflammatory cytokines in 24 patients. Spearman's correlation analysis revealed no statistically significant correlations between the SUVR values in any brain region and any serum cytokines (all *p* > 0.05) (Figure 5a). Given the limited sample size of this study, stringent significance thresholds might fail to detect potential but meaningful associations. However, even upon visual inspection of the scatterplots, no clear correlation trends were observed. Similarly, the hemispheric AI of [^18^F]DPA‐714 uptake showed no significant correlation with any serum marker (Figure 5b).

**FIGURE 5:**
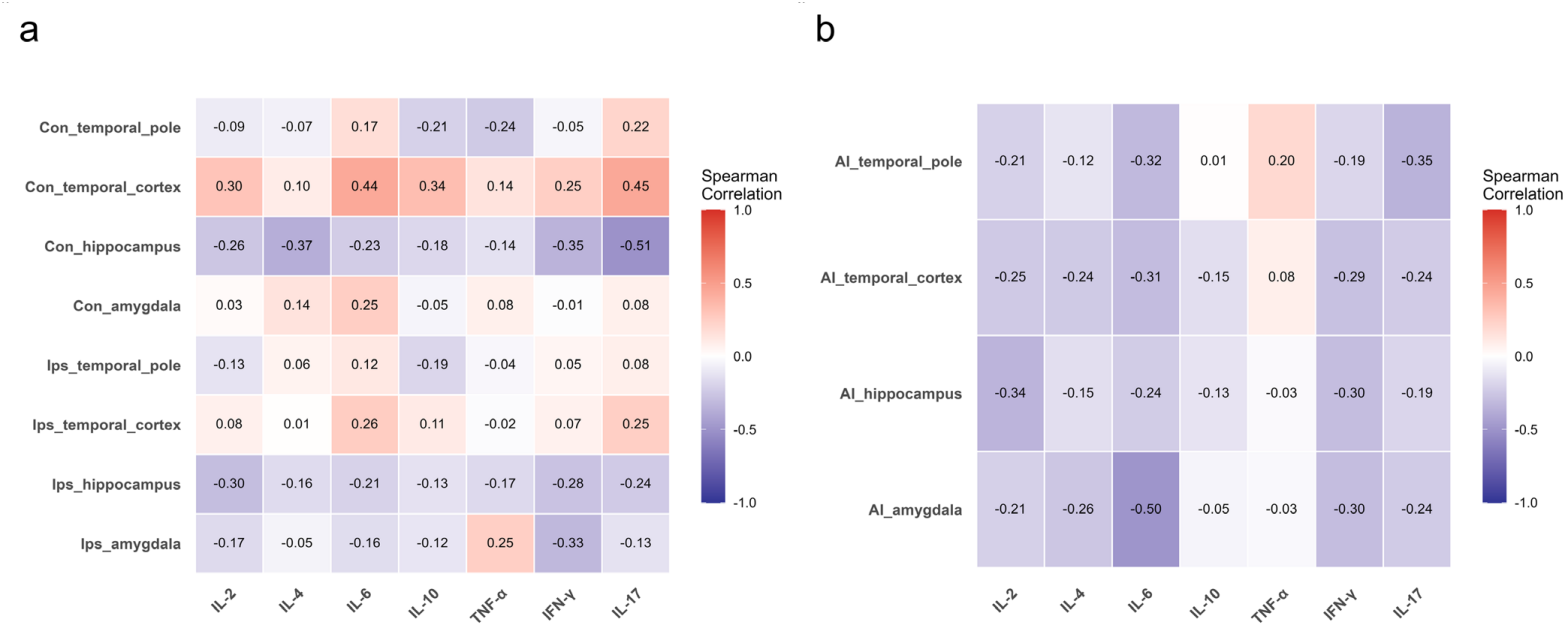
Correlation analysis of [^18^F]DPA‐714 SUVR and AI With cytokines. Heatmaps based on Spearman correlation analysis. (a) Correlations between SUVR in eight ROIs and cytokines. (b) Correlations between the AI of uptake in four brain regions and cytokines. All *p* values are uncorrected for multiple comparisons. AI was calculated as described. Color scale indicates Spearman's *ρ* values. AI, Asymmetry Index; ROIs, temporal regions of interest; SUVR, standardized uptake value ratio.

Given that simple correlation analysis may not adequately capture complex multivariate inflammatory states, we performed principal component analysis (PCA) on the serum inflammatory markers to reduce dimensionality and extract core components representing global peripheral inflammatory patterns. The analysis yielded two principal components (PC1 and PC2), which together accounted for 69.41% of the total variance. PC1 was primarily loaded by IL‐2, IL‐4, and TNF‐α (factor loading > 0.8), while PC2 was strongly correlated with IFN‐γ and IL‐17 (factor loading > 0.89) (Figure 6a). Statistical analysis revealed no significant correlation between the level of ipsilateral hippocampal [^18^F]DPA‐714 uptake and either peripheral inflammatory component: PC1 (*ρ* = −0.27, *p* = 0.20) or PC2 (*ρ* = −0.27, *p* = 0.20) (Figure 6b,c). To further assess the robustness of these findings, we performed partial correlation analyses adjusting for clinical covariates, including disease duration, seizure frequency, number of ASMs, drug resistance status, and seizure‐to‐scan interval. After adjustment, no significant correlations were observed between [^18^F]DPA‐714 uptake and the cytokine‐derived principal components (all adjusted *p* > 0.05; Figure S2).

**FIGURE 6:**
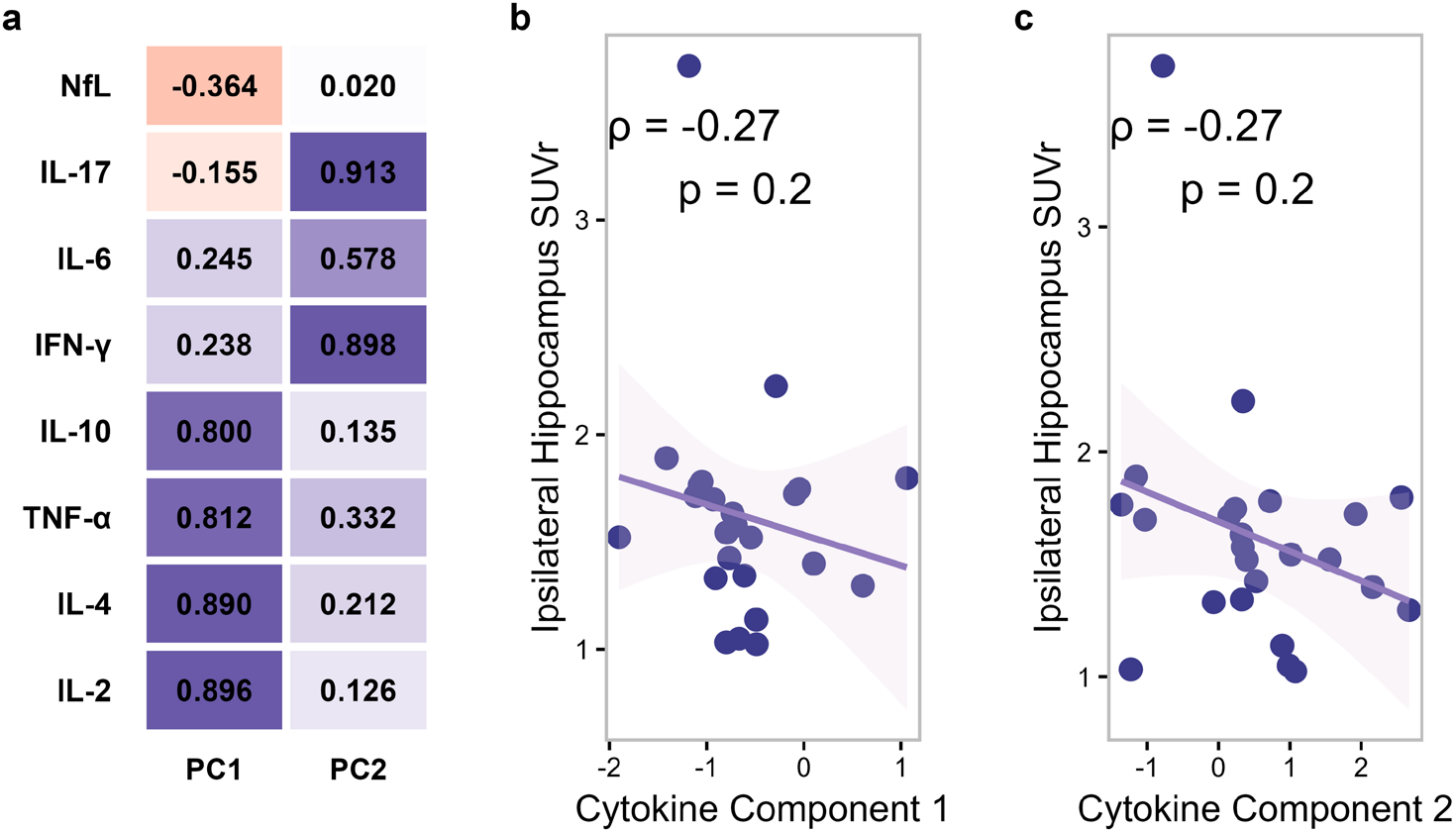
Associations between peripheral and central inflammation. (a) Contribution of each cytokine to Component 1 and Component 2; colors represent the strength and direction of the associations (b, c). Individual scores from cytokine‐derived Component 1 (b) and Component 2 (c) showed no significant correlation with [^18^F]DPA‐714 SUVR in the ipsilateral hippocampus (Spearman correlation analysis). All *p* values are uncorrected for multiple comparisons. SUVR, standardized uptake value ratio.

In summary, despite observing specific alterations in serum cytokines in patients with TLE, we found no significant correlation between serum cytokines and central neuroinflammation as quantified by neuroimaging.

## 4 Discussion

This study integrated [^18^F]DPA‐714 PET/MRI neuroimaging with peripheral serum cytokine profiling to investigate the central‐peripheral immune relationship in patients with TLE. Compared with HCs, TLE patients exhibited both increased [^18^F]DPA‐714 uptake in ipsilateral temporal lobe structures and altered serum cytokine levels, including elevated IL‐17 and IFN‐γ and decreased IL‐2, IL‐4, and IL‐10. However, no significant correlations were observed between [^18^F]DPA‐714 uptake in any temporal lobe region and any of the measured serum cytokines. This absence of correlation persisted across all eight temporal lobe regions of interest and all cytokine panels analyzed.

Quantitative [^18^F]DPA‐714 PET/MRI imaging demonstrated focally elevated [^18^F]DPA‐714 uptake within the ipsilateral temporal lobe in TLE patients. The observed pattern aligns with the results of prior research indicating that microglial activation, visualized via TSPO binding, serves as a key pathophysiological driver within epileptogenic networks [16]. Elevated [^18^F]DPA‐714 uptake not only serves as a marker for dynamic changes in microglia but also offers advantages in localizing the epileptogenic focus. In our cohort, it achieved a temporal lobe localization rate of 87.5% and a concordance rate of 78.95% with the gold‐standard combination of VEEG and MRI, both substantially higher than the rates obtained with [^18^F]FDG PET. These findings collectively suggest that [^18^F]DPA‐714 PET may provide complementary information for delineating the epileptogenic zone. In particular, for patients with drug‐resistant TLE who are MRI negative or who exhibit equivocal [^18^F]FDG‐PET results, [^18^F]DPA‐714 PET holds important potential for mapping the epileptogenic network [17, 18].

Through systematic multi‐cytokine analysis, our study revealed a characteristic immune shift in the serum of TLE patients: significant elevations in the levels of proinflammatory cytokines IFN‐γ and IL‐17, accompanied by synchronous reductions in the levels of anti‐inflammatory cytokines IL‐2, IL‐4, and IL‐10. The observed elevations in IFN‐γ and IL‐17 align with the recognized role of the Th1/Th17 pathway in epileptogenesis [6, 19], whereas the reduction in IL‐10 is consistent with the results of most studies focusing on TLE with hippocampal sclerosis [20, 21]. More significantly, this study clearly documents the marked suppression of IL‐2 and IL‐4—a broader immunoregulatory defect often overlooked in previous research [22]. Notably, the role of TNF‐α warrants particular consideration, although this classical proinflammatory cytokine, which is frequently reported to be elevated in TLE studies [23], did not differ significantly between the groups in standard analyses; PCA revealed that TNF‐α combines with IFN‐γ and IL‐17 to form a proinflammatory module, suggesting that these cytokines collectively represent a key inflammatory pathway in TLE pathogenesis. These findings suggest that in TLE, TNF‐α may not act in isolation but rather as part of a coordinated inflammatory network, in which its covariation with other mediators may be more biologically meaningful than its absolute concentration. Collectively, these cytokine patterns indicate that immune dysregulation in TLE involves both Th1/Th17 activation and Th2 response suppression, reflecting a comprehensive imbalance in T‐helper cell subsets [19, 24, 25].

Our study further revealed a significant association between central neuroinflammation and clinical severity in patients with TLE. Positive correlations were observed between ipsilateral hippocampal [^18^F]DPA‐714 uptake and both seizure frequency and epilepsy duration. The correlation with seizure frequency reinforces the concept of activity‐dependent neuroinflammation, whereby seizures directly amplify glial activation, potentially fostering a pro‐epileptogenic milieu, which is consistent with prior preclinical evidence [26]. More notably, the positive correlation with disease duration suggests that neuroinflammation accumulates over time in patients with TLE, potentially contributing to disease progression. This clinical correlation aligns with recent fMRI evidence of excitation–inhibition (E/I) imbalance in pharmaco‐resistant TLE, in which E/I ratio elevation correlated with disease duration and seizure frequency—a pattern that parallels our observed associations between TSPO uptake and clinical severity. Given that neuroinflammation modulates synaptic transmission and promotes neuronal hyperexcitability through glial activation and inflammatory mediators, the neuroinflammatory signal captured by TSPO PET and the E/I imbalance identified by resting‐state fMRI may represent complementary facets of the same underlying pathophysiological process in TLE progression [27]. These findings are inconsistent with those of some studies in focal cortical dysplasia (FCD), in which TSPO uptake was independent of disease duration [28]. This discrepancy may highlight distinct neuroinflammatory dynamics across epileptogenic etiologies: in TLE with hippocampal sclerosis, inflammation may be a core driver of progression, whereas in developmental lesions such as FCD, inflammation might represent a more “static” background process stemming from the inherent structural pathology. Collectively, our results position neuroinflammation as a key pathophysiological link between recent seizure activity and long‐term disease burden in patients with TLE, underscoring its potential as a therapeutic target to modify the disease course [4, 29].

The critical objective in this study was to investigate the link between central neuroinflammation and peripheral immune activity in patients with TLE. Despite clear alterations in both compartments, our analyses revealed no statistically significant correlations between serum cytokines (IFN‐γ, IL‐17, IL‐2, IL‐4, IL‐10, TNF‐α, or IL‐6) levels and the [^18^F]DPA‐714 SUVR values within any of the eight key temporal lobe ROIs. This lack of correlation persisted even when the AI of TSPO binding was used and after dimensionality reduction via PCA. This lack of correlation indicates that while peripheral and central inflammatory processes are both altered in TLE, they appear to operate, at least in the interictal state as assessed here, as relatively independent biological compartments. The lack of a simple, linear relationship between serum cytokine levels and focal brain TSPO binding underscores the complexity of neuroimmune interactions in epilepsy and suggests that peripheral cytokine profiles may not reliably serve as direct surrogates for localized brain inflammation in patients with TLE. Several pathophysiological factors specific to TLE may explain this finding. First, the relatively intact blood‐brain barrier during the interictal period may effectively isolate the brain's inflammatory milieu, preventing direct interactions between the periphery and the central immune system. Second, neuroinflammation in TLE is highly focal and network‐specific and primarily confined to the limbic system. This localized, chronic inflammatory process may not generate a signal robust enough to override the background noise of systemic immune activity. Third, the timing of assessment is critical. In our study, blood samples were collected at a median of 2 days (range: −4 to +8 days) relative to the PET/MRI scan. Although peripheral cytokine responses may be transient, studies have shown that TLE patients exhibit elevated cytokine levels in both postictal and interictal states, supporting the concept of chronic low‐grade inflammation [6, 30]. Therefore, the several‐day interval between blood collection and PET acquisition is unlikely to have substantially confounded our correlation analyses. Research on the link between peripheral and central inflammation in neurological diseases remains relatively limited and has yielded inconsistent results. Our data provide direct evidence for a lack of such correlation in interictal TLE, suggesting that this dissociation may represent a distinctive characteristic of the disease's chronic neuroinflammatory state.

The present study has several limitations that should be noted. First, although the sample size was sufficient for this initial multimodal exploratory study, it remains moderate and may thus limit statistical power to detect subtle associations—particularly in the complex interplay between peripheral and central inflammatory markers. Second, the cross‐sectional design precludes causal inferences regarding the relationship between neuroinflammation and seizure activity; longitudinal studies are needed to elucidate these dynamic interactions. Third, methodological variations in data acquisition should be noted. The interval between the last seizure and PET scanning differed across participants, and given the dynamic nature of postictal neuroinflammatory changes, this temporal variability could have influenced tracer uptake patterns. Finally, our assessment of the neuroimmune axis was largely confined to serum markers. Incorporating cerebrospinal fluid analysis and evaluating emerging communication pathways, such as the skull bone marrow–meningeal lymphatic system, could provide a more integrated understanding of central–peripheral immune dynamics and might help explain the observed dissociation between central and peripheral inflammation [31, 32].

In conclusion, this study demonstrated that focal [^18^F]DPA‐714 uptake is elevated in patients with TLE, and that ipsilateral hippocampal uptake is correlated with seizure frequency and disease duration, supporting a central role for neuroinflammation in disease progression. Serum cytokine profiling revealed a distinct immune imbalance in these patients, characterized by elevated proinflammatory cytokines and reduced anti‐inflammatory cytokines. However, we found no significant correlation between central and peripheral inflammatory measures, indicating that circulating cytokines may not be reliable surrogates for focal neuroinflammation in patients with TLE. These findings underscore the necessity of direct neuroimaging for assessing focal neuroinflammation in patients with TLE.

## Funding

This research was supported by the National Key Research and Development Program of China, no. 2022YFC2503804. National Natural Science Foundation of China, no. 82001234, no. 82271489, no. 81971203, no. 81801287. Science and Technology Program of Guangzhou, China, no. 2024A04J4564. Guangdong Provincial Clinical Research Center for Neurological Diseases, no. 2020B1111170002. Guangdong Province International Cooperation Base for Early Intervention and Functional Rehabilitation of Neurological Diseases, no. 2020A0505020004. Guangdong Provincial Engineering Center for Major Neurological Disease Treatment, Guangdong Provincial Translational Medicine Innovation Platform for Diagnosis and Treatment of Major Neurological Disease, and Guangzhou Clinical Research and Translational Center for Major Neurological Diseases.

## Disclosure

The authors have nothing to report.

## Ethics Statement

This study was conducted in accordance with the ethical standards of the Declaration of Helsinki and received approval from the Ethics Review Committee of the First Affiliated Hospital of Sun Yat‐sen University (Ethics Review [2024] No. 756‐1).

## Conflicts of Interest

The authors declare no conflicts of interest.

## Supporting information


**Figure S1:** Correlations between clinical severity measures and [^18^F]DPA‐714 SUVR in other ipsilateral temporal ROIs (amygdala, temporal cortex, and temporal pole). Spearman's *ρ* and *p* values are shown in each panel.

**Figure S2:** Partial correlation analyses between [^18^F]DPA‐714 SUVR and cytokine principal components adjusted for clinical covariates. Heatmaps showing partial correlation coefficients between [^18^F]DPA‐714 SUVR (asymmetry index of four regions: hippocampus, amygdala, temporal cortex, and temporal pole) and serum cytokine‐derived principal components (PC1 and PC2). (A) Partial correlations adjusted for disease duration, seizure frequency, number of ASMs and drug resistance status in the full valid sample (*n* = 20). (B) Partial correlations adjusted for seizure‐to‐scan interval in the subsample with complete interval data (*n* = 13). All *p* values were FDR‐corrected for multiple comparisons; no significant correlations were observed (all adjusted *p* > 0.05).

## Data Availability

The data that support the findings of this study are available from the corresponding author upon reasonable request.
